# The Level and Clinical Significance of B Lymphocytes and Immune Molecules (IL‐4, IL‐10, BAFF, and IgD) in Psoriasis

**DOI:** 10.1002/iid3.70413

**Published:** 2026-03-30

**Authors:** Boheng Wu, Ru Qin, Lichuan Lai, Shangyang Li, Ruilan Lin, Yunlong Zhang, Yao Guan, Yulin Yuan

**Affiliations:** ^1^ Graduate School Guangxi University of Chinese Medicine Nanning, Guangxi China; ^2^ Department of Laboratory Medicine 924th Hospital of the PLA Joint Logistics Support Force Guilin Guangxi China; ^3^ Department of Laboratory Medicine The People's Hospital of Guangxi Zhuang Autonomous Region, Joint training unit of Guangxi University of Chinese Medicine Nanning Guangxi China

**Keywords:** immune molecules, memory B cell, psoriasis, regulatory B cell

## Abstract

**Background:**

Psoriasis is an immune‐mediated chronic skin disease. Despite the low proportion of B cells in human blood, they play an important role in regulating the pathogenesis of psoriasis. Therefore, we investigated the role and clinical significance of B cells in psoriasis by conducting experiments.

**Materials and Methods:**

Thirty psoriasis patients and 30 healthy volunteers were selected as human subjects for skin biopsy collection and histological analysis, and EDTA anticoagulated blood was collected for flow cytometry and ELISA. The means of two independent samples were compared using an independent samples *t*‐test, and *p* < 0.05 was considered to be statistically significant.

**Results:**

Stained pathological sections from psoriasis patients revealed infiltration of a large number of cells in skin lesions. Flow cytometry and ELISA analysis revealed the following comparisons between psoriasis patients and healthy volunteers: significant upregulation of lymphocytes (*p* < 0.05); no significant difference in CD19+ B cells; significant difference in Bregs, CD19+ CD24+ CD38+ cells (*p* < 0.05); significant difference in memory B cells, CD19+ CD27+ CD38− cells (*p* < 0.01); significant difference in naive B cells, CD19+ CD27− CD38+ cells (*p* < 0.05); BAFF, IgD, and IL‐4 serum levels were much higher in PsO patients than those in healthy volunteers (*p* < 0.05). However, no remarkable difference in IL‐10 level (*p* > 0.05) was found.

**Conclusions:**

The levels of B cell populations as well as immune molecules including BAFF, IgD, and IL‐4 are significantly associated with psoriasis. These findings may lead to further investigations into the role of B cells and their subsets in the pathogenesis of psoriasis.

## 1 Introduction

Psoriasis is an autoimmune skin disease characterized by chronic inflammation [1, 2], raised, well‐defined erythematous papules, and silver‐scale plaques. Psoriasis can be of several types, including psoriasis vulgaris, pustular psoriasis, erythroderma psoriasis, and arthropathy psoriasis [3]. Among various factors that cause psoriasis, T cell‐mediated immune response is a popular one. Many studies have reported the relationship between psoriasis and T cells [4, 5], but studies on the mechanism of action and clinical significance of B cells in psoriasis are rare. However, some studies have shown that B cells and specific B‐cell subsets can negatively regulate the immune response in mice, suggesting the presence of Bregs [6]. Therefore, this study focused on B cells in psoriasis and evaluated the level of expression and clinical significance of B cells and their subpopulations in psoriasis.

B‐cells are derived from hematopoietic stem cells, a process that is under complex regulation [7]. B cells are important components of the adaptive immune system [8]. They can secrete antibodies and act as an important bridge to promote the occurrence and development of immune‐related diseases [9]. Although they represent only 1%–7% of leukocytes [10, 11], B cells play important roles in immune defense, including immunoglobulin production, antigen presentation, and cytokine secretion [9, 12]. Bregs are immunosuppressive cells that play important roles in immune tolerance. They account for less than 10% of circulating B cells in healthy individuals and regulate immune tolerance and homeostasis by secreting interleukin (IL)‐10, IL‐15, and TGF‐β [13]. Bregs also inhibit the differentiation of Th 1 cells and Th 17 cells [14, 15]. To determine the relationship between B cells and psoriasis, whole‐blood samples from 30 psoriasis patients and healthy volunteers were collected, flow cytometry was performed to determine the proportion of B lymphocytes in the blood and the serum content of B cell cytokines (BAFF, IgD, IL‐4, and IL‐10) was determined by Enzyme‐Linked Immunosorbent Assay (ELISA). Percentage of B cell populations and cytokine levels of psoriasis patients and healthy volunteers were analyzed using FlowJo and GraphPad Prism 8.0.

## 2 Materials and Methods

### 2.1 Human Subjects

From January 2023 to 2024, 30 psoriasis patients hospitalized in the outpatient department of the People's Hospital of Guangxi Zhuang Autonomous Region, including 20 males and 10 females aged 16–78 years, with an average age of 42 ± 16 years, were included. The diagnostic criteria were as follows: a history of other autoimmune diseases, blood diseases, tumors, liver and kidney diseases, and acute and chronic infections. Additionally, 30 healthy volunteers from the Health Examination Center of the People's Hospital of Guangxi Zhuang Autonomous Region were selected; including 11 males and 18 females aged from 22 to 45 years, and the average age was 34 ± 6 years. The above clinical data were collected following the ethical standards set by the Human Trial Committee, and informed consent was obtained from the subjects.

### 2.2 Materials

The BD FACS Canto II flow cytometer (BD Biosciences), monoclonal antibodies against CD38‐APC (Lot:25019942) and CD19‐PE‐Cy7 (Lot:2626679), and red cell lysis buffer Hybri‐Max (Lot:RNBM0102) were purchased from Thermo Fisher. CD24‐PE (Lot: CD024220801) and CD27‐PerCP Cy5.5 (Lot:4064205) were purchased from BD Biosciences. BAFF, IgD, IL‐4, and IL‐10 levels were quantified using commercial ELISA Kit (FineTest) were purchased from Upingbio.

### 2.3 Methods

#### 2.3.1 Skin Biopsy Collection and Histological Analysis

A skin biopsy was performed by a dermatologist, who separated 2–3 cm of psoriatic lesion tissue from a patient with psoriasis and diagnosed it as a psoriatic skin lesion. Normal skin biopsies were collected from healthy volunteers, fixed, embedded, cut into thin sections as found for psoriatic skin, and stained with hematoxylin and eosin (H&E). All images were evaluated under a microscope (Olympus BX53).

#### 2.3.2 Sample Collection

All participants fasted for 12 h, and 3 mL of EDTA anticoagulant was collected between 8:00 a.m. and 9:00 a.m. Within 30 min, samples were rapidly centrifuged at 3000 rpm for 5 min and then serum samples were collected and stored at −80°C immediately until initiated ELISA. Sample after centrifugation were stored at room temperature and analyzed by flow cytometry within 6 h of collection.

#### 2.3.3 Flow Cytometry and ELISA Assay

To conduct flow cytometry, 50 μL of anticoagulated whole blood and 5 μL of CD19‐PE Cy7, CD38‐APC, CD27‐PerCP Cy5.5, and CD24‐PE were mixed. After incubating at room temperature for 15 min, 500 μL of hemolysin was added, mixed, and incubated for 10–12 min at room temperature. After dissolving the red blood cells, they were centrifuged at 1500 rpm for 5 min, and the supernatant was discarded. The cells were washed once with 2 mL of phosphate‐buffered saline (PBS) and centrifuged at 1500 rpm for 5 min. The supernatant was discarded, and the cells were resuspended in 300 μL of PBS to produce the desired single‐cell suspension. BAFF, IgD, IL‐4, and IL‐10 levels were quantified using commercial ELISA Kit (FineTest). The correlation coefficient *r* value of calibrator dose‐response curve is greater than or equal to 0.9900, the intra‐batch coefficient of variation CV% of kit is less than 10%, and the inter‐batch coefficient of variation CV% is less than 15%. Sample diluent wells and blank wells are set as quality control during the experiment. All assays were conducted according to manufacturer's protocols.

#### 2.3.4 Statistical Analysis

The data of flow cytometry were analyzed using FlowJo and GraphPad Prism 8.0. The data of ELISA were analyzed using GraphPad Prism 8.0. The means of two independent samples were compared using an independent samples *t*‐test, and *p* < 0.05 was considered to be statistically significant.

## 3 Results

### 3.1 Skin Biopsy Collection and Histological Analysis

**Figure 1:**
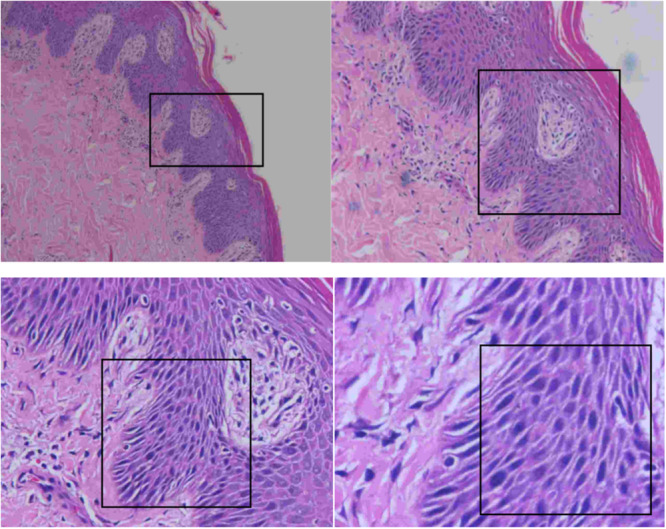
Representative images of H&E‐stained psoriatic skin biopsies. Formalin‐fixed paraffin‐embedded sections were stained with H&E. Scale bar = 100 µm. H&E, hematoxylin and eosin.

Representative images of H&E‐stained skin sections from normal and psoriasis patients are shown in Figure 1. Psoriatic skin had extensive hyperkeratosis and confluent keratosis compared to normal skin. The epidermis was hyperplastic, with squamous epithelium pseudoepithelial hyperplasia, superficial keratosis, disappearance of the focal granular layer, slightly spongy, dermal papillary edema with vascular dilation, and infiltration of perivascular lymphocytes and plasma cells. Changes in the skin epidermis of psoriasis patients may impair the barrier function of the skin.

### 3.2 Gating Strategy

**Figure 2:**
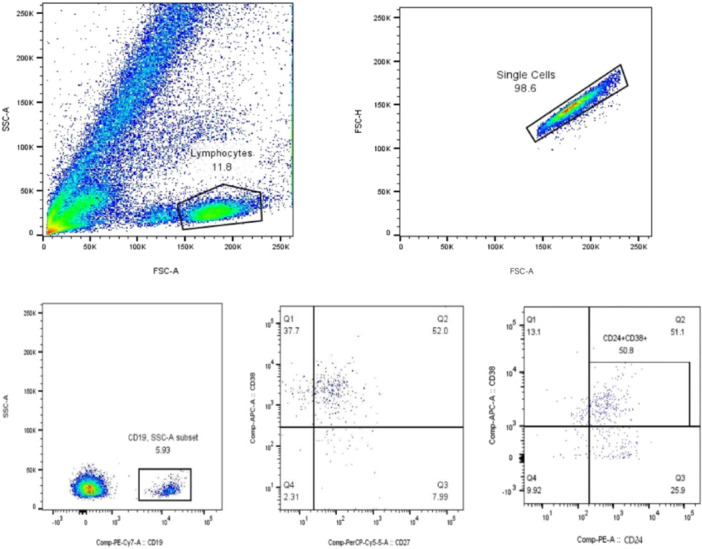
CD27/CD38 and CD24/CD38 gating strategies. The CD24, CD27 and CD38 expression gating strategy divided circulating B cells into CD27+ CD38− (memory), CD24+ CD38+ (Bregs), and CD38+ CD27− (naive) cells.

Flow cytometry data were analyzed using FlowJo software. The gating strategy was as follows: lymphocytes were first gated on an FSC‐A/SSC‐A plot to exclude debris, followed by doublet exclusion (FSC‐A/FSC‐H). Total B cells were identified as CD19+ cells. From this population, B‐cell subsets were characterized based on CD27, CD38, and CD24 expression using Fluorescence Minus One (FMO) controls to define positivity. The definitions were: naive B cells (CD19+ CD27− CD38+), memory B cells (CD19+ CD27+ CD38−), and Bregs (CD19+ CD24+ CD38+). The distribution of these subsets on CD38/CD27 and CD38/CD24 axes is shown in Figure 2.

### 3.3 Statistical Analysis

#### 3.3.1 The Data of Flow Cytometry

##### 3.3.1.1 Lymphocyte and B Cell Counts

**Figure 3:**
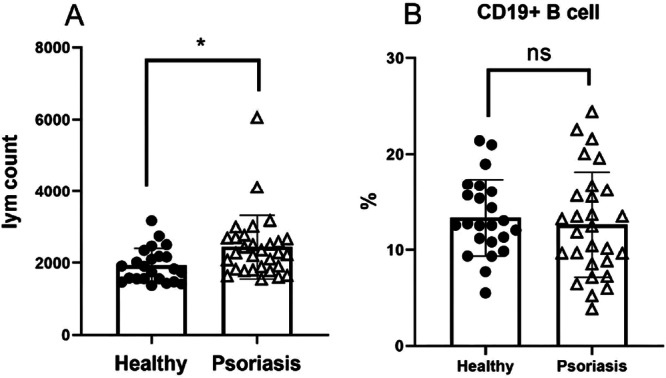
Frequency of lymphocytes and B cells in the peripheral blood of psoriatic patients and healthy controls. For the frequency of lymphocyte count (A), significant differences were found between the sample means (*p < 0.05). The frequency of total CD19+ B cells (B) was not significantly different between the sample means.

As shown in Figure 3A, the absolute number of lymphocytes was significantly higher in psoriasis patients (2439.60 ± 885.93 cells/µL) compared to healthy controls (1952.52 ± 477.63 cells/µL), with a mean difference of 487.08 cells/µL (95% CI: 96.45–877.71; *t* = 2.51, *p* = 0.015). However, the percentage of CD19+ B cells within the lymphocyte gate did not differ significantly between patients (12.61% ± 5.49%) and controls (13.34% ± 4.01%; mean difference: −0.73%, 95% CI: −3.49 to 2.03; *t* = −0.53, *p* = 0.599) (Figure 3B).

##### 3.3.1.2 B Cell Subset Distribution

**Figure 4:**
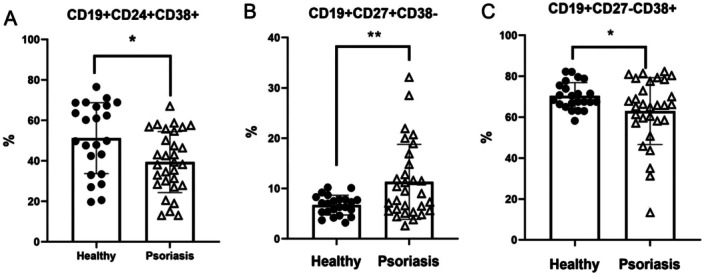
The frequency of B‐cell subsets in the peripheral blood of patients with psoriasis and healthy controls. For CD19+ CD24+ CD38+ Bregs (A) and CD19+ CD27− CD38+ naive B cells (C), significant differences were found between the sample means (*p < 0.05). For CD19+ CD27+ CD38− memory B cells (B), significant differences were found between the sample means (**p < 0.01).

Analysis of B cell subpopulations revealed significant alterations in psoriasis patients compared to controls (Figure 4A–C). After applying FDR correction for multiple comparisons, the following differences were observed.

Regulatory B cells (Bregs): The frequency of Bregs (CD19+ CD24+ CD38+) was significantly lower in psoriasis patients (39.39% ± 15.07%) than in healthy controls (51.22% ± 17.54%), with a mean difference of −11.83% (95% CI: −20.83 to −2.83; *t* = −2.65,*p* = 0.011, Cohen's *d* = 0.72), indicating a medium‐to‐large effect.

Naive B cells: The proportion of naive B cells (CD19+ CD27− CD38+) was also decreased in patients (62.92% ± 16.31%) compared to controls (70.33% ± 6.51%; mean difference: −7.41%, 95% CI: −14.63 to −0.19; *t* = −2.07, *p* = 0.045, Cohen's *d* = 0.56), representing a medium effect size.

Memory B cells: Conversely, the frequency of memory B cells (CD19+ CD27+ CD38−) was significantly higher in the psoriasis group (11.33% ± 7.43%) than in the control group (6.68% ± 1.96%; mean difference: 4.65%, 95% CI: 1.53–7.77; *t* = 3.01, *p* = 0.005, Cohen's *d* = 0.82), indicating a large effect size.

#### 3.3.2 The Data of ELISA

**Figure 5:**
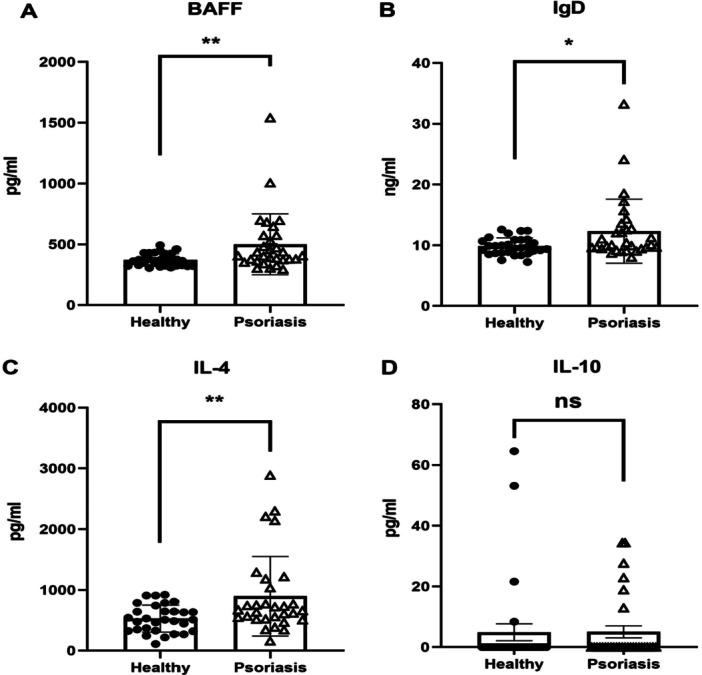
B cell cytokines including BAFF (A), IgD (B), IL‐4 (C), and IL‐10 (D) in PsO patients (*n* = 30) and healthy volunteers (*n* = 30). Comparisons between the two groups were made using an unpaired *t*‐test, with error bars representing the mean ± SEM. ns, not significant; **p < 0.01; *p < 0.05.

We further compared the serum concentrations of B‐cell‐related cytokines between psoriasis patients and healthy controls (Figure 5A–D). After applying FDR correction for multiple comparisons across the four measured factors, the following differences were observed

BAFF: Serum BAFF levels were significantly elevated in psoriasis patients (499.95 ± 202.71 pg/mL) compared to healthy controls (259.23 ± 129.93 pg/mL), with a mean difference of 240.72 pg/mL (95% CI: 66.38–415.06; *t* = 2.81, *p* = 0.008, Cohen's *d* = 1.38), indicating a very large effect size.

IgD: Patients with psoriasis exhibited significantly higher IgD levels (12.30 ± 5.27 µg/mL) than controls (9.83 ± 1.4 µg/mL; mean difference: 2.47 µg/mL, 95% CI: 0.49–4.45; *t* = 2.53, *p* = 0.016, Cohen's *d* = 0.63), representing a medium‐to‐large effect.

IL‐4: Serum IL‐4 concentrations were markedly increased in the psoriasis group (895.31 ± 654.50 pg/mL) relative to the control group (527.57 ± 222.77 pg/mL), with a mean difference of 367.74 pg/mL (95% CI: 120.38–615.10; *t* = 3.02, *p* = 0.005, Cohen's *d* = 0.74), indicating a large effect size.

IL‐10: In contrast, no significant difference was observed in IL‐10 levels between psoriasis patients (5.04 ± 10.86 pg/mL) and healthy controls (4.92 ± 15.31 pg/mL; mean difference: 0.12 pg/mL, 95% CI: −6.64 to 6.88; *t* = 0.04, *p* = 0.972, Cohen's *d* = 0.01).

## 4 Discussion

Psoriasis can be of several types, including psoriasis vulgaris, pustular psoriasis, erythroderma psoriasis, and arthropathy psoriasis [3]. Because the dermatology department of the author's hospital did not perform PASI score for psoriasis and the evaluation data of disease severity were incomplete, this study did not analyze the correlation between results and disease severity. The samples collected in this study were outpatients, and most of them were follow‐up visits to psoriasis vulgaris, so the results of this study are representative. In the future, the correlation between disease severity and study results will be fully considered when designing experiments, and the clinical data of included cases will be improved in cooperation with clinical departments, and the results will be analyzed among psoriasis subtypes. In this study, H&E‐stained psoriasis pathological sections were used to observe cell infiltration, and extensive hyperkeratosis and confluent keratosis, which are classic manifestations of psoriatic skin lesions, were detected in the skin lesions of psoriasis patients. The histology of psoriatic plaques is usually acanthosis (epidermal hyperplasia), which involves an inflammatory infiltrate composed of dermal dendritic cells, macrophages, T cells, and neutrophils [16]. Additionally, we also examined the difference between B cells in psoriasis patients and healthy people in whole blood by flow cytometry and reported that the total lymphocyte number significantly increased in psoriasis patients (*p* < 0.05), but the difference in B‐cell frequency suggested that for different psoriasis types and disease development stages, the number of B cells may be impaired in the immune regulation stage or that psoriasis patients receiving different degrees of treatment have fewer B‐cells. The proportion of CD19+ B cells was significantly greater in the PBMCs of patients with psoriasis vulgaris and arthropathic psoriasis than in those of healthy controls, which was also reported by Lu et al. [17]. The percentage of CD19+ B cells was significantly lower in the PBMCs of patients with erythroderma psoriasis and pustular psoriasis than in those of healthy controls. However, the proportion of CD19+ B cells in lesions was greater than that in non‐diseased patients. These findings indicated that the proportion of CD19+ B cells may be positively associated with the PASI score in patients with psoriasis vulgaris. In another study, B‐cell levels were lower in PsA patients than in controls. After treatment with etanercept, the number of B cells increased significantly [18].

We also found that Breg cells in psoriasis whole blood were significantly downregulated (*p* < 0.05), This may be related to the fact that the cases collected in this study were follow‐up cases where biological agents were used to suppress the immune response. Treatment with anti‐IL‐17A (ixekizumab) and anti‐TNF‐α (adalimumab) significantly downregulates the numbers of T cells, macrophages, and dendritic cells in skin lesions, bringing them close to levels observed in healthy skin. However, the immune cell profile in non‐lesional areas does not fully return to normal [19]. Vildagliptin topical treatment effectively corrected histological abnormalities in psoriasis and significantly attenuated the overexpression of inflammatory markers (TNF‐α, IL‐17A, IL‐23, and IL‐22), angiogenic markers (VEGF), oxidative stress components (MDA and SOD), and proliferation factors (Ki‐67) [20]. This strongly indicates that both inflammatory factors and immune cells in psoriasis are affected following treatment. In a study, the blood of CD19+ CD24hi CD38hi B10 progenitors was compared to that of healthy controls, and the proportion of IL‐10‐producing Bregs (B10 cells) was found to be lower in psoriasis patients than in healthy controls. Additionally, the number of B10 progenitor cells was greater in psoriasis patients than in healthy controls, and fewer regulatory B10 cells producing IL‐10 were detected, suggesting that B10 cells may be impaired in psoriasis patients [21]. In our experiments, IL‐10 content in psoriasis serum was similarly minimal and showed no significant variability compared to healthy subjects. Although Bregs are functionally defined by their capacity to produce IL‐10, we did not observe a significant difference in serum IL‐10 levels between the groups. Several possible explanations may account for this finding: First, the immunoregulatory function of Bregs is not solely dependent on IL‐10 but may also involve TGF‐β or cell‐contact‐dependent mechanisms [22]; Second, serum IL‐10 levels may primarily originate from other cellular sources (such as Tregs or macrophages) and may not accurately reflect the functional status of local Bregs; Third, a recent study by Kitano et al. demonstrated that although the frequency of IL‐10+ Bregs is reduced in psoriasis patients, this change does not necessarily translate into altered circulating IL‐10 levels, and the recovery of Breg function may require longer timeframes or specific microenvironmental signals [23]. Therefore, the reduced frequency of Bregs may indicate impaired regulatory capacity, rather than simply leading to decreased serum IL‐10 levels. Yanaba et al. studied 60 patients with psoriatic arthritis, 50 psoriatic patients, and 23 healthy volunteers and found that CD19 (+) CD27 (+) CD24 (high) and CD19 (+) CD24 (high) CD38 (high) Bregs were lower in psoriatic patients with psoriatic arthritis and psoriasis [24]. B10 cells were inversely associated with the number of IL‐17A + CD3 + T cells and IFN‐γ + CD3 + T cells. In psoriasis patients, the number of IL‐10‐producing B cells was also negatively correlated with the PASI score [25, 26]. As an anti‐CD20 monoclonal antibody, rituximab can deplete B cells. A study reported that some patients developed psoriasis after treatment with rituximab [27]. However, rituximab also has a therapeutic effect in patients with psoriasis [28, 29]. Overall, these studies suggested that Breg subsets may play a negative role in the pathogenesis of psoriasis, especially in B10 cells. Moreover, B10 cells may inhibit the differentiation of IL‐17A‐ and IFN‐γ‐producing T cells. However, these T cells play a key role in psoriasis. Given that the multiple stages of B‐cell differentiation and the specific markers of B10 cells are not fully defined, further studies are needed to clarify the role of B cells in the pathogenesis of psoriasis. Therefore, more effort is required to improve the disease stage of psoriasis and maintain detailed records of patient cases, which is also a limitation of these experiments. In this experiment, we also found that memory B cells CD19+ CD27+ CD38− in psoriasis were significantly upregulated, while mature naive B cells CD19+ CD27− CD38+ were significantly downregulated. These findings suggested that mature naive B cells differentiate into memory B cells at the onset of psoriasis. Berkowska et al. reported that healthy controls presented normal numbers of blood IgE (+) plasma cells and normal numbers of CD27 (+) IgE (+) memory B cells but a higher number of CD27 (−) IgE (+) memory B cells compared to psoriatic patients [30]. The number of memory B cells also differed among different antibody types in psoriasis, which needs to be further investigated via experimental studies.

The ELISA data showed that the levels of BAFF, IgD, and IL‐4 in the serum of psoriasis patients were significantly higher than those in healthy individuals, but there was no significant difference in IL‐10 levels. B cells participate in immune responses through various cytokines, including BAFF. BAFFR is expressed on the surface of all peripheral B cell subpopulations and is known for its role in the survival and maturation of B cells. Its expression is upregulated in response to the tense BCR signal expression of the functional B cell antigen receptor (BCR), which enhances the expression of BAFFR in immature and transitional B cells [31, 32, 33]. In this experiment, the level of BAFF in psoriasis serum was significantly higher than that in healthy individuals (*p* < 0.01) (Figure 5A). This may suggest that BAFF drives B cells to participate in the inflammatory response of psoriasis. BAFF also plays an important role in T cell activation in T cell‐mediated diseases. Jacob et al. [34] found that subjects with autoimmune diseases such as lupus and Sjogren's syndrome had elevated levels of BAFF. In addition, IL‐4, IL‐10, and IgD can also affect the involvement of B cells in inflammatory responses. Our results indicate that the levels of IL‐4 and IgD in psoriasis serum are significantly higher than those in healthy individuals and have differences, but there is no significant difference in IL‐10 levels in psoriasis (Figure 5B–D). Abbas et al.'s finding that effective treatment elevates IL‐10 provides critical context for our observation of reduced Bregs (CD19+ CD24+ CD38+) in psoriasis patients. While we did not observe significant differences in serum IL‐10 levels, we now discuss that Bregs exert regulatory functions through multiple mechanisms (including cell‐contact dependent suppression and TGF‐β production) [35], and that serum IL‐10 may primarily originate from other cellular sources such as Tregs and macrophages. The reduced Breg frequency likely reflects impaired regulatory capacity rather than simply decreased IL‐10 production. IL‐4 is a key cytokine that drives the differentiation of Th2 cells that produce IL‐4 from naive T cells. In addition to Th2 cells, eosinophils, mast cells, NK T cells, and type II innate lymphocytes have been shown to produce IL‐4 [36, 37, 38]. Cascella et al. [39] evaluated the region by detecting 1526 subjects (500 PsA, 426 PsV, 600 controls), selecting SNPs of interest through real‐time PCR and direct sequencing, and genotyping them. The results were analyzed using biostatistics and bioinformatics, and KIF3A and interleukin‐4 were described as new PsA susceptibility genes, indicating the clear meaning of bone metabolism genes in the pathogenesis of diseases. IL‐10 is produced by certain subsets of monocytes, Th2 cells, mast cells, activated T cells, and B cells [40]. IL‐10 is an effective inhibitor of monocyte/macrophage function, inhibiting the production of many pro‐inflammatory cytokines, including TNF ‐ α, IL‐1 β, IL‐6, MIP‐1 α, and IL‐8, despite increased release of MCP‐1 [41, 42]. In the experiment, there was no significant difference in the level of IL‐10 in psoriasis serum compared to healthy individuals, which may be due to the inhibition or impaired function of Bregs. The main purpose of this experiment is to detect the levels of immune molecules IL‐4, IL‐10, and IgD in psoriasis, and to understand the role and clinical significance of B cells in psoriasis through these immune molecules.

## 5 Conclusion

This study focused on the level of B‐cell types in the whole blood of psoriasis patients and investigated the variability between Bregs and memory B cells and other classified B cells in the blood of psoriasis patients and healthy controls. By conducting experiments, we detected Bregs in the whole blood of psoriasis patients, memory B cells CD19+ CD27+ CD38− and CD19+ CD27− CD38+ in mature naive B cells. The experimental results suggested that the function of Bregs in psoriasis may be impaired and reduced and that naive B cells may differentiate into memory B cells after stabbing. The ELISA data showed that the levels of BAFF, IgD, and IL‐4 in the serum of psoriasis patients were significantly higher than those in healthy individuals, but there was no significant difference in IL‐10 levels. These immune molecules and B cells are of significant importance in the inflammatory process of psoriasis. However, the specific immune factors involved in the response of B cells need to be confirmed by more specific experiments. The limitation of this experimental study is that the course and severity of psoriasis were not evaluated. Due to the lack of PASI assessment for psoriasis patients in clinical diagnosis, correlation analysis between the disease and test data cannot be conducted. We will pay attention to this issue and design more detailed experimental studies in the future. To summarize, B cells play an important role in the pathogenesis of psoriasis, and our study revealed the relationship between B cells and psoriasis. The study can provide clinical value and research ideas for the mechanism of B cells in psoriasis.

## Author Contributions

**Yulin Yuan:** initiated and designed the research. **Yulin Yuan** and **Boheng Wu:** performed the research and wrote the original draft. **Boheng Wu** and **Ru Qin:** prepared samples and performed data analysis and interpretation. **Lichuan Lai**, **Shangyang Li**, **Ruilan Lin**, **Yunlong Zhang**, and **Yao Guan:** contributed to essential reagents and tools and supervised the study. All authors contributed to writing, reviewing, and editing the manuscript. All authors have read and approved the final version of the manuscript.

## Ethics Statement

This study was approved by the ethics committee of The People's Hospital of Guangxi Zhuang Autonomous Region (Ethics No. KY‐KJT‐2021‐83). Informed written consents were obtained from all participants prior to the commencement of the study.

## Conflicts of Interest

The authors declare no conflicts of interest.
